# Fatigue in Patients with Idiopathic/Isolated REM Sleep Behavior Disorder

**DOI:** 10.3390/brainsci12121728

**Published:** 2022-12-17

**Authors:** Yajie Zang, Hui Zhang, Yuan Li, Yanning Cai, Jagadish K. Chhetri, Piu Chan, Wei Mao

**Affiliations:** 1 Department of Neurology, Xuanwu Hospital of Capital Medical University, Beijing 100053, China; 2 Department of Neurobiology, Xuanwu Hospital of Capital Medical University, Beijing 100053, China; 3 Department of Biobank, Xuanwu Hospital of Capital Medical University, Beijing 100053, China; 4 National Clinical Research Center for Geriatric Disorders, Xuanwu Hospital of Capital Medical University, Beijing 100053, China; 5 Key Laboratory for Neurodegenerative Diseases of the Ministry of Education, Beijing 100053, China

**Keywords:** fatigue, iRBD, prevalence, depression, Parkinson’s disease

## Abstract

Introduction: Fatigue is one of the most common and disabling symptoms of Parkinson’s Disease (PD). The occurrence and clinical features of fatigue in patients with prodromal PD remain largely elusive. This study aimed to investigate the prevalence and clinical characteristics of fatigue in patients with idiopathic/isolated REM sleep behavior disorders (iRBD). Methods: A total of 97 polysomnography-confirmed iRBD patients were enrolled in this study. A comprehensive neurological assessment (including motor and non-motor assessment) was performed. Fatigue was assessed using the Fatigue Severity Scale (FSS). Motor and non-motor characteristics were compared between iRBD patients with and without fatigue. Logistic regression was used to identify the factors associated with fatigue. Results: The prevalence of fatigue was 35.05%. Compared to the non-fatigue patients, patients with fatigue had higher non-motor symptom scale (NMSS) score (*p* = 0.009), higher Hamilton Depression Rating Scale (HAMD) score (*p* = 0.002), and a higher prevalence of orthostatic hypotension (*p* = 0.021). Multivariate regression analysis showed that depression (OR 4.17, 95% CI 1.13–15.49, *p* = 0.033) and orthostatic hypotension (OR 2.80, 95% CI 1.09–7.18, *p* = 0.032) were significantly associated with fatigue in iRBD patients. Additionally, both NMSS (r_s_ = 0.310, *p* = 0.002) and HAMD (r_s_ = 0.385, *p* < 0.001) scores were mildly correlated with fatigue severity. Conclusion: Our study showed that fatigue is common in patients with iRBD. In addition, depression and orthostatic hypotension were independently associated with fatigue in iRBD patients.

## 1. Introduction

Fatigue is generally defined as a persistent sense of tiredness or exhaustion unexplained by any pre-existing medical conditions [1]. In neurological illnesses, fatigue has been described as a difficulty in initiating or maintaining focus on voluntary activities or a feeling of disconnection between expended effort and attempted activities [2].

Fatigue is one of the most common non-motor symptoms in patients with Parkinson’s Disease (PD) [3]. Fatigue has been suggested to be a contributor to poor quality of life and disability in PD [3]. Previous studies reported a varying prevalence of fatigue in patients with PD, with estimates ranging from 33% to 58% [4,5,6]. Fatigue can be present in the early phase of PD and is reported to occur even in the 2 to 10 years premotor period [7]. Yet, there is a limited understanding of the prevalence and clinical characteristics of fatigue in prodromal PD.

Idiopathic/isolated REM sleep behavior disorder (iRBD) is a parasomnia characterized by dream-enacting behaviors associated with the loss of normal muscle atonia in REM sleep on polysomnography [8]. Previous longitudinal studies have shown that patients with iRBD have an increased risk of developing neurodegenerative diseases, in particular α-synucleinopathies such as PD, dementia with Lewy bodies, and multiple system atrophy [9,10]. Therefore, iRBD is now recognized as a prodromal feature of PD and other α-synucleinopathies [8,11].

Several biomarkers of neurodegeneration in iRBD have been identified, including clinical symptoms and signs of subtle parkinsonism motor changes, hyposmia, constipation, impaired color vision, and neurocognitive impairment [8]. However, to our knowledge, no study has investigated the presence of fatigue in iRBD patients. To design therapeutic strategies for slowing down the progression of PD, a better understanding of symptoms such as fatigue in the prodromal phase of PD may be meaningful. Therefore, we conducted a cross-sectional study to estimate the prevalence of fatigue in iRBD. We further investigate the relationship of fatigue with motor and non-motor symptoms in patients with iRBD.

## 2. Materials and Methods

### 2.1. Study Participants

Patients aged >50 years diagnosed with iRBD were consecutively enrolled from the Department of Neurology, Xuanwu Hospital of Capital Medical University, between October 2012 and April 2021. All patients underwent overnight video-polysomnography (vPSG) in the sleep laboratory of Xuanwu hospital and met the diagnostic criteria for iRBD based on the standard International Classification of Sleep Disorders criteria (ICSD-III) [12]. Exclusion criteria included: (1) a history of mental disorders making the participants difficult to comply with the study; (2) diagnosed history of parkinsonism or dementia; (3) currently suffering from any medical conditions that may lead to fatigue.

All participants underwent detailed neurological evaluations to exclude any symptoms or signs of dementia or parkinsonism. Three subjects with anemia, congestive heart failure, and hypothyroidism were excluded. A total of 97 vPSG-confirmed iRBD patients were included in the final analysis.

The ethics committee of the Xuanwu Hospital, Capital Medical University, reviewed and approved this study. All enrolled subjects or their representatives gave written informed consent to participate in this study.

### 2.2. Clinical Assessment

We used the Fatigue Severity Scale (FSS) to quantitatively measure the severity of fatigue in iRBD patients. The FSS is a 9-item self-administered fatigue rating scale assessing the severity of fatigue symptoms [13]. FSS includes items on the physical, mental, and social aspects of fatigue. These are rated on a seven-grade Likert scale where 1 = strongly disagree to 7 = strongly agree. The final score is the sum of all item scores divided by 9. Participants were categorized according to the FSS score into two groups: fatigue (FSS ≥ 4.0) and non-fatigue (FSS < 4.0) [14].

The severity of RBD symptoms was evaluated using the RBD questionnaire–Hong Kong (RBDQ-HK) [15], and the duration of the RBD symptoms was recorded. To further analyze the relationship between fatigue and nocturnal dream-enactment behaviors in iRBD, the scores of factor two subscale of RBDQ-HK (Q6–Q11, behavioral/dream-enactment factor) were calculated. Clonazepam taken at the time of assessment was recorded. Motor symptoms were measured using part III of the Movement Disorder Society United Parkinson’s Disease Rating Scale (MDS-UPDRS) [16]. Quantitative motor testing was performed using Purdue Pegboard test [17]. Purdue Pegboard is a test of motor speed, hand dexterity, and finger-eye coordination. Subjects were given 30 s to transfer a series of pins from a dish, one at a time, into corresponding holes. This was performed separately in each hand, and the number of pins placed by the dominant and non-dominant hands was used as the outcome measure.

The non-motor Symptom Scale (NMSS) was used to assess the overall non-motor symptoms burden [18]. Both Montreal Cognitive Assessment (MoCA) and Mini Mental-Status Examination (MMSE) were used to assess global cognitive function [19]. We used Hamilton Depression Rating Scale (HAMD) to assess the mood state of the participants, and depression was defined as a score of HAMD ≥ 8 [20]. Apathy was screened by the part IA of MDS-UPDRS. The apathy item is scored on a 5-point scale from 0–4, with a score of 0 categorized as non-apathy and a score > 0 grouped as apathy. Participants were also evaluated for excessive daytime sleepiness (EDS) with Epworth Sleepiness Scale (ESS), with EDS defined as an ESS score ≥ 10 [21]. Constipation was defined using the Rome III criteria [22], and insomnia was diagnosed based on the ICSD-III criteria [12]. Olfactory function was assessed by a 12-item Brief Smell Identification Test (B-SIT), with hyposmia defined as a score < 8 [23]. Subjects with anosmia due to a history of nasal surgery or severe nasal diseases did not complete the B-SIT. Orthostatic hypotension (OH) was defined as a decrease in systolic blood pressure (SBP) of ≥20 mmHg and/or a decrease in diastolic blood pressure (DBP) of ≥10 mmHg while changing the posture from supine to the upright position within 3 min [24].

### 2.3. Statistical Analysis

All continuous variables were presented as mean ± standard deviation (SD), and categorical variables as counts and percentages. The normal distribution of variables was assessed with the Shapiro–Wilk normality test. Comparison between groups (i.e., Fatigue vs. Non-fatigue) was performed with regard to the demographic and clinical variables. Comparison between groups was performed using the student t-test for normally distributed continuous variables, Mann–Whitney U test for non-normally distributed continuous variables, and chi-square test for categorical variables.

Univariate and multivariate logistic regression models were used to investigate the factors associated with fatigue. The presence or absence of fatigue was set as the dependent variable in the model. Variables with *p* < 0.1 in the univariate analysis (except for the total NMSS) and the variables considered clinically related to fatigue in previous studies in the general population (i.e., age and gender) were entered into the multivariate logistic regression analyses model. Multicollinearity in the regression model was assessed using an examination of the variance inflation factor (VIF). The Correlations between the FSS score and other clinical variables in the total sample were analyzed with Pearson’s or Spearman’s correlation coefficients for parametric or non-parametric variables, respectively. All *p*-values were two-tailed, and *p*-values < 0.05 were considered to be statistically significant. All statistical analyses were performed using the SPSS software (SPSS Version 25.0, IBM Corp., Armonk, NY, USA).

## 3. Results

### 3.1. Baseline Demographics and Clinical Characteristics

According to the suggested cut-off score in FSS, the prevalence of fatigue in iRBD patients was 35.05% (34/97).

Table 1 shows the comparison of baseline demographic characteristics and clinical features of fatigued and non-fatigued patients in iRBD. Compared to the non-fatigue group, the total score of the NMSS was significantly higher in the fatigue group (42.18 ± 27.55 points vs. 25.97 ± 14.36 points, *p* = 0.009). The fatigue group had a significantly higher HAMD score (6.85 ± 7.50 points vs. 2.65 ± 3.21 points, *p* = 0.002) and a higher proportion of depression (29.4% vs. 7.9%, *p* = 0.005) than the non-fatigue group. Four patients were taking α1-receptor blockers or vasodilators at the time of assessment, so their blood pressure data were excluded from the final analysis. In the remaining 93 patients, we found that the proportion of patients with OH was significantly higher in the fatigue group compared to the non-fatigue group (66.7% vs. 41.7%, *p* = 0.021). However, no significant difference was observed in the UPDRS-III score, Purdue Pegboard test scores, RBDQ-HK total score, and factor two subscale score, MMSE, and MoCA score between the groups. We also observed no differences in the prevalence of insomnia, EDS, hyposmia, constipation, and clonazepam use between the two groups. The fatigue group showed a higher proportion of apathy than the non-fatigue group but without statistical significance (*p* = 0.069).

### 3.2. Factors Associated with Fatigue

Univariate logistic regression analysis showed that a high NMSS score (OR 1.04, 95% CI 1.02–1.06), high HAMD score (OR 1.19, 95% CI 1.06–1.32), depression (OR 4.83, 95% CI 1.49–15.64), and OH (OR 2.80, 95% CI 1.15–6.80) were associated with fatigue in iRBD (Table 1). Age, gender (male vs. female), OH (presence vs. absence), depression (presence vs. absence), and apathy (presence vs. absence) were included in the multivariate logistic regression model. The multivariate logistic regression analysis showed that depression (OR 4.17, 95% CI 1.13–15.49, *p* = 0.033) and OH (OR 2.80, 95% CI 1.09–7.18, *p* = 0.032) were significantly associated with fatigue in patients with iRBD (Table 2).

### 3.3. Correlation of Fatigue Severity with Motor and Non-Motor Symptoms in iRBD

Bivariate correlation analyses of the patients with iRBD are presented in Table 3. We found that the FSS score had positively weak associations with the HAMD score (r_s_ = 0.385, *p* < 0.001) and the NMSS score (r_s_ = 0.310, *p* = 0.002). There were no significant correlations between the FSS score with the UPDRS-III score, Purdue Pegboard test score, MMSE, MoCA, and ESS score.

## 4. Discussion

To our knowledge, this is the first study to investigate the prevalence and characteristics of fatigue in patients with iRBD. Our study showed that fatigue was a common presentation in patients with iRBD, with an estimated prevalence of 35.05%. We found that a high NMSS score, OH, and depression were associated with higher odds of fatigue in iRBD patients. Furthermore, our study also showed both depression and OH to be independently associated with higher odds of fatigue in iRBD patients. Additionally, the scores of NMSS and HAMD had weak correlations with the severity of fatigue.

Previous studies on fatigue in the general population showed a prevalence ranging from 18% to 25% [25,26]. The prevalence of fatigue was reported to be between 37% to 58% in patients with PD [4,5,6]. Our study reported the prevalence of fatigue to be 35.05% in patients with iRBD, which was slightly lower than that in patients with PD but much higher than the prevalence of fatigue in the general population. This result indirectly supports the theory that fatigue may potentially appear in the very early phase of PD and could even manifest in the premotor stage of the disease [7]. Given the reported negative impact of fatigue on quality of life [3], our data highlighted the importance of assessing fatigue in patients with iRBD.

It is worth noticing that we found a small (albeit not significant) difference in age between the fatigue and non-fatigue groups in iRBD. The previous meta-analysis had reported that patients with fatigue had slightly higher age (approximately one year older) compared to the non-fatigued patients in PD [27]. However, previous communities-based studies showed that fatigue was strongly associated with aging in the general population [28,29]; a possible reasonable explanation was that older people presented more diminished physical functioning and a higher tendency to seek medical attention in comparison to the younger population [30]. In fact, the importance of fatigue is so crucial in the aging population that it has been considered a major component of frailty- a common geriatric condition that makes an individual vulnerable to stressors [31]. A previous study showed fatigue in community-dwelling older adults was associated with the APOE4 genotype [32]. Future studies on the relation between fatigue and iRBD should also explore such genetic association. 

Our study showed that fatigue in patients with iRBD was closely related to depression. Previous studies also suggested that PD patients with coexisting depression had a significantly higher risk of developing fatigue [4,33,34]. Considering that the presence of fatigue is one of the clinical symptoms of major depression in the DSM criteria, there is a significant overlap between these two clinical conditions. However, the findings that successful treatment of depression did not constantly improve symptoms of fatigue in PD, suggesting that fatigue and depression might be two distinct entities. It is well known that noradrenergic and serotonergic system dysfunction is related to depression [35]. Interestingly, it is reported that PD patients with fatigue had a greater decrease in serotonin transporter binding capacity in the basal ganglia and limbic structures [36] and a lower concentration of serotonergic metabolites in CSF compared to those without fatigue [37]. This suggests that serotonergic system dysfunction might also contribute to the presence of fatigue in PD. The overlapping pathophysiological mechanisms involving serotonergic projections in basal ganglia and limbic circuits may explain the close association between fatigue and depression. Future studies will be needed to further investigate the pathophysiological mechanism of fatigue in iRBD using molecular imaging and pathology. Additionally, it would be worth prospectively following up with the iRBD patients to assess whether fatigue persists after comorbid depressive symptoms resolve.

The association between fatigue and OH has been previously reported in PD as well as in MSA [38,39]. Likewise, our results found a similar association between fatigue and OH in patients with iRBD. One possible explanation is that patients with OH often complain of fatigue, generalized weakness, dizziness, titubation, blurry vision, syncope, nausea, and falls due to temporary cerebral hypoperfusion and reflexive sympathetic hyperactivity [40]. Whether OH is involved in the pathophysiological mechanism of fatigue in iRBD remains unknown. Interestingly, a novel hypothesized degeneration route in PD involves a pathological process originating from the enteric nervous system with subsequent caudo-rostral propagation to the autonomic and central nervous system, potentially causing a clinical phenotype (i.e., body-first PD) dominated by RBD, fatigue, and autonomic symptoms—in particular OH [24]. Hence, further study is needed to identify the nature and potential mechanisms of the association between fatigue and OH in iRBD.

Our study showed that patients with fatigue had a higher total NMSS score compared to the non-fatigue group, and there was a weak positive correlation between NMSS scores and FSS scores in our patients. It suggests that the presence of fatigue was associated with a moderate burden of non-motor symptoms. However, we did not observe a significant relationship between motor symptoms (i.e., UPDRS-III and Purdue Pegboard test score) and fatigue or between cognitive performance and fatigue, which indicates that fatigue in iRBD does not likely present as a result of prodromal parkinsonism motor change or cognitive deterioration. Although we supposed that patients with more frequent dream enacting behaviors might have more nighttime sleep disruptions, which may contribute to daytime fatigue. However, this study found no significant relationship between dream-enactment behaviors and fatigue in patients with iRBD.

We observed that iRBD patients with fatigue had a higher proportion of apathy than the non-fatigued ones, although the difference did not achieve statistical significance. This finding is similar to the results in patients with PD, as described by a previous meta-analysis that patients with fatigue were moderately more apathetic than those without fatigue in PD [27]. It was likely partly caused by the lack of uniform definitions of apathy and fatigue. Both apathy and fatigue are multidimensional concepts, and their various subdimensions might be differently related to each other and overlap to some extent. In addition, the similarity between apathy and fatigue might be explained by a common neurobiological basis. It had been proposed that the disturbances in the prefrontal-basal ganglia loop might be the underlying mechanisms for both apathy and fatigue [41]. Both symptoms are related to the degeneration of serotoninergic pathways and abnormal activity and connectivity of limbic-cortical circuits in PD [41]. It should also be noted that the symptom of apathy was assessed by a single question in MDS-UPDRS part IA in our study. Future studies should include more objective apathy proxies to widely investigate the relationship between apathy and fatigue in patients with iRBD.

This is the first study investigating the prevalence and related factors of fatigue in iRBD patients using a relatively large sample size and comprehensive clinical assessments. However, several limitations of this study should be acknowledged. First, our analysis used available MDS-UPDRS data for the determination of apathy. While the MDS-UPDRS scale for the determination of apathy has been used before, it relies on a single item with no objective grading. The lack of a standardized interview and assessment for the evaluation of apathy is a potential limitation. Second, although we comprehensively evaluated the nonmotor symptoms in our patients, we did not assess anxiety with a detailed rating scale such as Hamilton Anxiety Rating Scale. Therefore, the relationship between fatigue and anxiety could not be explored. Third, fatigue as a feeling is difficult to define; therefore, medication history, especially clonazepam use, is one of the major confounding factors that could impact the feeling of fatigue in iRBD. However, we did not find a significant difference in the use of clonazepam between the patients with fatigue and non-fatigue. Future studies are needed to investigate this issue. Fourth, we did not include a group of healthy controls. Thus, the characteristics of fatigue in patients with iRBD and the normal older populations could not be compared. Fifth, our analyses related to the early expression of fatigue were based on a cross-sectional study. Thus, a causal relationship cannot be established from this study. Finally, the utilization of a uni-dimensional fatigue measure was another limitation, considering fatigue is a multidimensional symptom. Since the data collection started before the recently recommended diagnostic criteria for fatigue proposed by Kluger and colleagues [42], it was impossible to use the new recommendation in this study. Moreover, it should be noted that the FSS scale is the most commonly used fatigue scale and the only scale to receive “recommended” ratings from the Movement Disorders Society Task Force for screening and measuring severity.

## 5. Conclusions

Fatigue is a common symptom in patients with iRBD, with an estimated prevalence of 35.05%. A high NMSS score, depression, and the presence of OH were associated with fatigue in iRBD. Unified and multidimensional assessment tools and large-scale multicenter studies are needed in the future to attain a better understanding of fatigue in iRBD patients.

## Figures and Tables

**Table 1:** Baseline clinicodemographic characteristics and univariate analyses of fatigue in patients with iRBD.

Clinical Characteristics	Fatigue (*n* = 34)	Non-Fatigue (*n* = 63)	*p* Value	Univariate Analysis	
				OR (95% CI)	*p* Value
Age, years	68.41 ± 1.39	66.49 ± 0.85	0.216 *	1.04 (0.98–1.10)	0.214
Male, *n* (%)	26 (76.5)	47 (74.6)	0.839	1.11 (0.42–2.93)	0.839
RBDQ-HK score	57.47 ± 16.48	52.62 ± 18.05	0.368	1.02 (0.99–1.04)	0.196
RBD duration, years	7.68 ± 5.41	6.49 ± 6.47	0.162	1.03 (0.97–1.10)	0.365
RBDQ-HK subscale score (Factor 2)	28.12 ± 11.87	26.17 ± 10.40	0.688 *	1.02 (0.98–1.06)	0.413
UPDRS-III score	1.91 ± 2.22	2.12 ± 2.16	0.532	0.95 (0.78–1.16)	0.640
Purdue Pegboard test					
dominant hand score	13.45 ± 1.64	13.51 ± 1.60	0.865 *	0.98 (0.75–1.27)	0.865
non-dominant hand score	13.07 ± 1.69	12.87 ± 1.80	0.588 *	1.07 (0.84–1.36)	0.588
Total score of NMSS	42.18 ± 27.55	25.97 ± 14.36	0.009	1.04 (1.02–1.06)	0.001
MMSE score	28.12 ± 1.63	27.87 ± 1.72	0.538	1.09 (0.85–1.41)	0.493
MoCA score	24.56 ± 2.78	24.21 ± 2.96	0.738	1.04 (0.90–1.21)	0.565
HAMD score	6.85 ± 7.50	2.65 ± 3.21	0.002	1.19 (1.06–1.32)	0.002
Depression, *n* (%)	10 (29.4)	5 (7.9)	0.005	4.83 (1.49–15.64)	0.009
ESS score	6.18 ± 3.46	5.38 ± 3.56	0.157	1.07 (0.95–1.20)	0.290
EDS, *n* (%)	4 (11.8)	9 (14.3)	0.972	0.80 (0.23–2.82)	0.728
Apathy, *n* (%)	16 (47.1)	18 (28.6)	0.069	2.22 (0.93–5.29)	0.071
Insomnia, *n* (%)	17 (50.0)	27 (42.9)	0.500	1.33 (0.58–3.08)	0.501
OH, *n* (% ) (*n* = 93)	22 (66.7)	25 (41.7)	0.021	2.80 (1.15–6.80)	0.023
B-SIT score (*n* = 90)	6.80 ± 1.99	7.10 ± 2.47	0.471	0.95 (0.78–1.14)	0.560
Hyposmia, *n* (%)	17 (56.7)	32 (53.3)	0.765	1.14 (0.47–2.77)	0.765
Constipation, *n* (%)	27 (79.4)	47 (74.6)	0.595	1.31 (0.48–3.59)	0.596
Clonazepam use, *n* (%)	10 (29.4)	13 (20.6)	0.332	1.60 (0.62–4.17)	0.334

* Student t-test. Abbreviations: RBD: REM sleep behavior disorder; RBDQ-HK: RBD questionnaire-Hong Kong; UPDRS-III: Unified Parkinson’s Disease Rating Scale-III; NMSS: Non-Motor Symptom Scale; MMSE: Mini Mental-Status Examination; MoCA: Montreal Cognitive Assessment; HAMD: Hamilton Depression Rating Scale; ESS: Epworth Sleepiness Scale; EDS: excessive daytime sleepiness; OH: orthostatic hypotension; B-SIT: Brief Smell Identification Test.

**Table 2:** Multivariate logistic regression to identify the factors associated with fatigue.

Variables	B Value	OR (95% CI)	*p* Value
Age	0.035	1.04 (0.97–1.11)	0.295
Male	0.176	1.19 (0.38–3.76)	0.764
OH	1.030	2.80 (1.09–7.18)	0.032
depression	1.429	4.17 (1.13–15.49)	0.033
Apathy	0.698	2.01 (0.75–5.42)	0.167

Abbreviations: OR: odds ratio; CI: confidence interval; OH: orthostatic hypotension.

**Table 3:** Correlations between the FSS score and clinical characteristics.

Variables	Correlation Coefficient	*p* Value
UPDRS-III score	−0.080	0.439
Pegboard test score (dominant hand)	−0.003 *	0.977
Pegboard test score (non-dominant hand)	0.036 *	0.723
MMSE score	0.011	0.913
MoCA score	0.045	0.662
NMSS score	0.310	0.002
HAMD score	0.385	<0.001
ESS score	0.059	0.568

* Pearson Correlation coefficient. Abbreviations: UPDRS-III: Unified Parkinson’s Disease Rating Scale-III; MMSE: Mini Mental-Status Examination; MoCA: Montreal Cognitive Assessment; NMSS: Non-Motor Symptom Scale; HAMD: Hamilton Depression Rating Scale; ESS: Epworth Sleepiness Scale.

## Data Availability

Data collected for the study are available upon reasonable request.
